# Long-Term Outcomes of Birdshot Chorioretinopathy Treated with Corticosteroids: A Case Reports

**DOI:** 10.3390/jcm12165288

**Published:** 2023-08-14

**Authors:** Dino Ferracci, Thibaud Mathis, Antoine Gavoille, Mathieu Gerfaud-Valentin, Arthur Bert, Meriem Hafidi, Philippe Denis, Olivier Loria, Laurent Kodjikian, Pascal Sève

**Affiliations:** 1 Department of Ophthalmology, Croix-Rousse University Hospital, Hospices Civils de Lyon, 69004 Lyon, France; 2 UMR5510 MATEIS, CNRS, INSA Lyon, Université Lyon 1, 69100 Villeurbanne, France; 3 Department of Biostatistics-Bioinformatics, Hospices Civils de Lyon, 69007 Lyon, France; 4 Department of Internal Medicine, Hôpital de la Croix-Rousse, Hospices Civils de Lyon, 69004 Lyon, France; 5 Hospices Civils de Lyon, Pôle IMER, 69003 Lyon, France; 6 University Lyon, University Claude Bernard-Lyon 1, HESPER EA 7425, 69008 Lyon, France

**Keywords:** birdshot chorioretinopathy (BSCR), corticosteroids, inflammatory macular edema, prognosis, relapse, uveitis

## Abstract

**Highlights:**

**What is known?**

- Corticosteroids alone are considered ineffective in treating and preventing relapses in birdshot chorioretinopathy (BSCR).
- Although many studies have been conducted in recent years, highlighting the effectiveness of immunosuppressive drugs, there is currently no consensus on the optimal treatment modalities and duration of treatment for BSCR.
- We have previously shown in a series of 14 patients that intravenous corticosteroid therapy followed by prednisone treatment resulted in control of ocular inflammation in 71% of patients.

**What is new?**

- The prolonged corticosteroid therapy treatment strategy resulted in inflammation control in almost half of our patients with BSCR. This control was maintained with low doses of cortisone, usually less than 5 mg daily.
- Initial loading doses did not appear to significantly reduce the time needed to reach inflammation control or the number of relapses, nor did they improve the final visual prognosis. However, we could not conclude that methylprednisolone may not be helpful since the two groups were different at baseline, with more patients with macular edema treated with initial intravenous corticosteroids.

**Abstract:**

Purpose: To report the progression of patients diagnosed with birdshot chorioretinopathy (BSCR) initially treated with corticosteroids. Methods: We included 39 BSCR patients that were followed for ≥1 year. We analyzed their progression under treatment after 1, 3, 6 months, 1 year, and at the end of follow-up. In order to determine the efficiency of initial loading doses, patients were classified into two groups according to their initial treatment: methylprednisolone followed by prednisone (n = 28) and prednisone alone (n = 11). Results: At the end of follow-up, 31/39 (79.5%) patients had reached inflammation control. Thirteen out of 28 (46.4%) and 6/11 (54.5%) patients were treated exclusively with corticosteroids, and 18/19 (94.7%) of them had reached inflammation control at the end of follow-up; their mean (range) corticosteroid dose was 3.5 (0–10) mg/day. Conclusions: We found that the prolonged corticosteroid therapy treatment strategy resulted in inflammation control in half of BSCR patients. This control was maintained with low doses of cortisone, usually <5 mg/day.

## 1. Introduction

Birdshot chorioretinopathy (BSCR) is a rare, bilateral, chronic uveitis that affects mostly Caucasian patients, generally aged over 50. It is responsible for approximately 1% of uveitis cases and 8% of posterior segment uveitis [1]. It is a pure ophthalmological entity presumed to be autoimmune [2], resulting in both choroidal inflammation in the form of primary stromal choroiditis and retinal inflammation in the form of vasculitis, papillitis, and/or inflammatory macular edema (IME) [3]. Campimetric alterations, reduced contrast and color vision, myodesopsia, and visual blur are the most frequent symptoms, although objective visual acuity is often preserved for a long time [4,5,6].

Originally described by Ryan and Maumenee in 1980 [7], then rapidly redefined by Gass in 1981 [8], the clinical and paraclinical characterization of this condition has evolved considerably over the last forty years. There are now established diagnostic criteria based on genetic data and multimodal imaging, particularly in indocyanine green angiography (ICGA) and fluorescein angiography (FA) [9,10,11]. Thanks to these new findings, BSCR can now be diagnosed and treated before the appearance of the depigmented “cream-colored” lesions described by Gass [8], thereby considerably improving the patient’s visual prognosis.

BSCR is associated with the HLA A29 antigen in almost all cases [10], which represents the strongest known association of a disease with a histocompatibility complex. The presence of HLA-A29 has become an essential element in the diagnosis of BSCR according to the latest SUN classification [12]. Some authors, therefore, propose a new and more appropriate terminology for this condition, namely “HLA-A29 retinochoroidopathy” [10].

BSCR treatment has not been evaluated in controlled studies and various approaches have been discussed for the care of patients. As for noninfectious bilateral uveitis in general, systemic corticosteroids remain the mainstay of the therapy due to their rapid anti-inflammatory and immunosuppressive effects [6,13,14]. The treatment of BSCR with corticosteroids solely is considered ineffective [15,16] and the early introduction of second-line immunosuppressive treatment (e.g., cyclosporin, mycophenolate mofetil, methotrexate) has been advocated to minimize the visual loss and the side effects related to high doses of corticosteroids [17]. More recently, adalimumab and tocilizumab have shown their effectiveness in refractory BSCR cases [18,19,20].

A series issued by our research team including 14 patients has previously shown that intravenous corticosteroid therapy followed by prednisone treatment (mean dose of 6.2 mg/day prednisone) resulted in the control of the ocular inflammation in 71% of patients one year after treatment initiation [21]. In the present study, we reported the long-term outcome of patients initially treated for BSCR with intravenous then oral corticosteroids, or with solely oral corticosteroids, in two tertiary uveitis centers.

### 1.1. Patients and Methods

We conducted a retrospective analysis of medical records from patients with a clinical diagnosis of BSCR in two university hospital centers (Hôpital de la Croix-Rousse, Lyon, France and Hôpital Edouard Herriot, Lyon, France) between 1 January 2005 and 31 January 2022. All patients diagnosed with BSCR defined by the SUN working group in its latest version [12], treated with corticosteroids, and followed for at least 1 year were included. Patients who had received previous treatment and whose inflammatory course, visual acuity, relapse, or treatment tolerance data were not available were excluded. The study received approval from the local ethics committee in February 2019 (No. 19–31) and was registered on clinicaltrials.gov (NCT03877575).

### 1.2. Data Collection

Each patient medical record was retrospectively reviewed in order to collect demographic data: age at the initial visit, gender, ethnicity, medical history, and presence of HLA-A29 allele. The dates of the first visit, the date of BSCR diagnosis, previous and concomitant treatment, and follow-up duration were also recorded.

The following ophthalmologic characteristics were collected at diagnosis and during follow-up: current visual symptoms, best corrected visual acuity (BCVA) assessed by Snellen charts, anterior segment examination (intraocular pressure, slit lamp biomicroscopy), indirect ophthalmoscopy, vitreous inflammation reaction quantified as described by Nussenblatt et al. [22], and presence of vasculitis. Indocyanine angiography was principally used for diagnosis and for monitoring choroiditis (when available). Fluorescein angiography was used for monitoring vasculitis (defined as retinovascular leakage), papillitis, and macular edema, when available. The ocular coherence tomography images evaluated macular thickness and macular edema.

Regarding the treatments used, data about indication, dosage, route of administration, duration of use, and cause of suspension were collected. Side effects were also recorded.

The loading doses of methylprednisolone varied between 5 and 10 mg/kg administered over a period of three days. In analogy with giant cell arteritis, high dose gluco-corticoid therapy (40–60 mg/day prednisone-equivalent) was immediately used [23,24]. Prednisone tapering, with or without an initial loading dose, consisted of a target dose of 15–20 mg/day within 2–3 months and after 1 year to ≤5 mg/day. The taper below 5 mg/day was adapted for each patient according to his or her profile (relapser, initial inflammatory macular edema…).

### 1.3. Clinical Assessment

We analyzed the progression under treatment after 1, 3, 6 months, 1 year, and at the end of the follow-up. To evaluate the effectiveness of each treatment, the control of inflammation was defined by the following criteria: absence of intraocular inflammation at the slit lamp examination and indirect ophthalmoscopy, resolution of vasculitis observed on fluorescein angiogram (chronic vasculitis reflecting a disruption of the inner blood-retinal barrier was tolerated), and absence of macular edema [25].

Thus, at each visit, we assessed the inflammation control according to the presence of an improvement or a worsening, allowing us to determine whether the inflammation was controlled or not.

Patients were grouped into two groups according to their initial treatment: the first group was composed of patients treated with methylprednisolone pulse followed by oral prednisone (methylprednisolone group), and the second group was composed of patients treated with oral prednisone alone (prednisone group).

### 1.4. Outcome Measures

The outcome measures were (1) change in the best corrected visual acuity (BCVA), (2) time to inflammation control, (3) number of relapses and time to relapse, and (4) use of an immunosuppressive drug. We also followed the progression of patients with inflammatory macular edema (IME) at first examination or who developed IME during the follow-up. The safety of cortisone and cortisone-sparing treatments was also investigated.

### 1.5. Statistical Analysis

Continuous variables were expressed as mean ± standard deviation (SD; BCVA), mean or median and range (treatment doses/follow-up duration), or median [interquartile range, IQR] (other variables), and categorical variables were expressed as count (percentage). Visual acuity was transformed into logMAR and analyzed using a linear mixed-effect model, with time (before or after treatment) and treatment group as fixed-effects, and an eye effect nested within a patient effect as random intercept to account for intra-patient correlation when studying both eyes of a same patient. The Gaussian distribution of the random effect and residuals was visually assessed using a quantile-quantile plot. The annualized relapse rate according to treatment group was analyzed in a negative binomial model to correct the over-dispersion, with the logarithm of the follow-up duration as offset. The use of an immunosuppressive drug was compared between treatment groups using the Fisher exact test. The time to inflammation control was represented by a cumulative incidence curve using the Kaplan–Meier approach and compared between treatment groups using the log-rank test. The time to relapse was compared between treatment groups using the Wilcoxon rank test. Finally, comparison of the inflammation control in subgroups with/without IME was performed using a Fischer exact test. *p*-values less than 0.05 were considered statistically significant. Analyzes were performed using R software, version 4.0.3 (R Core Team (2020). R: A language and environment for statistical computing, R Foundation for Statistical Computing, Vienna, Austria).

## 2. Results

### 2.1. Demographics

**Figure 1:**
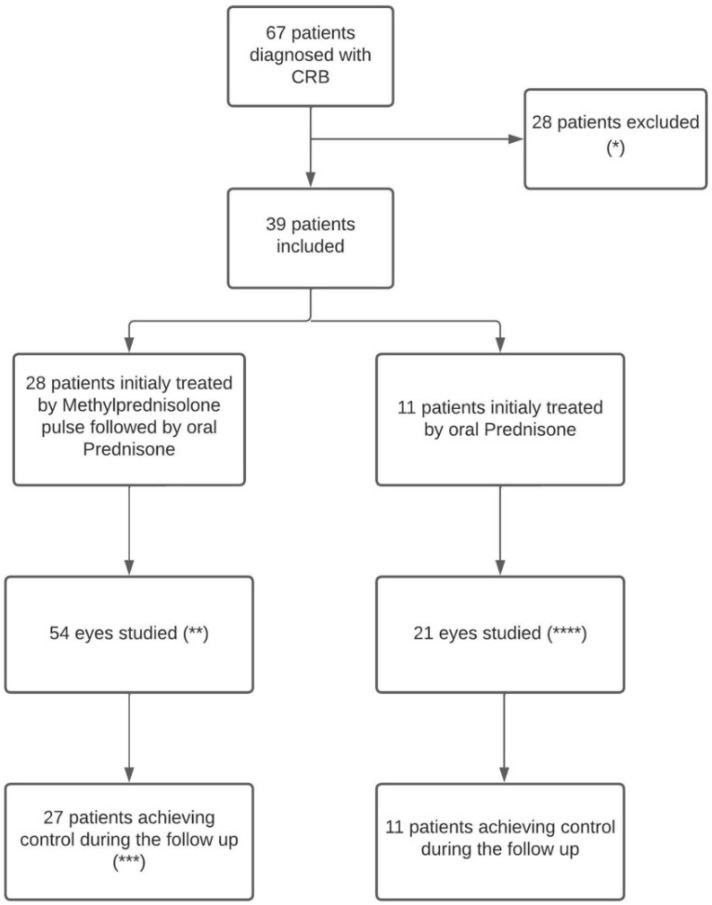
Flowchart. BCVA: best-corrected visual acuity. (*) Three patients were excluded because they did not meet the SUN diagnostic criteria [12], 23 because their follow-up duration was <1 year, or because of missing data, and two because their initial treatment was not a corticosteroid. (**) BCVA value at end of follow-up was not available for two eyes from the same patient, excluding those from the BCVA analysis. (***) One patient never achieved inflammation control in the methylprednisolone group, excluding him from various analysis including time to control, number of relapses, and time to relapse. (****) One patient had a single functional eye.

Of the 67 patients diagnosed with BSCR and followed, 39 patients met the inclusion criteria and were, therefore, included in the present study. The remaining 28 patients were excluded because of the follow-up duration was <1 year or because of missing data (n = 23), two because their initial treatment was not a corticosteroid, and three patients were excluded because they did not meet the SUN diagnostic criteria [12]. A total of 28 patients were treated with initial methylprednisolone pulse and 11 patients with oral prednisone only (Figure 1). All patients were Caucasian and HLA A-29 positive, and 20 (51.3%) were women. The mean (range) delay from symptom onset to diagnosis was 17.9 (1–120) months. The mean (range) follow-up duration was 78.8 (23–186) months. The mean (range) patient age was 50 (26–79) years. The mean ± SD initial BCVA (LogMar) was 0.122 ± 0.192. IME was present in 20 (26.0%) eyes corresponding to 13 (33.3%) patients (Table 1). There was a significant difference in BCVA between eyes with IME (mean: 0.262) and those without IME (mean: 0.045; mean difference: 0.217; (95% CI [0.136; 0.297]; *p* < 0.001)).

### 2.2. BCVA Progression

**Figure 2:**
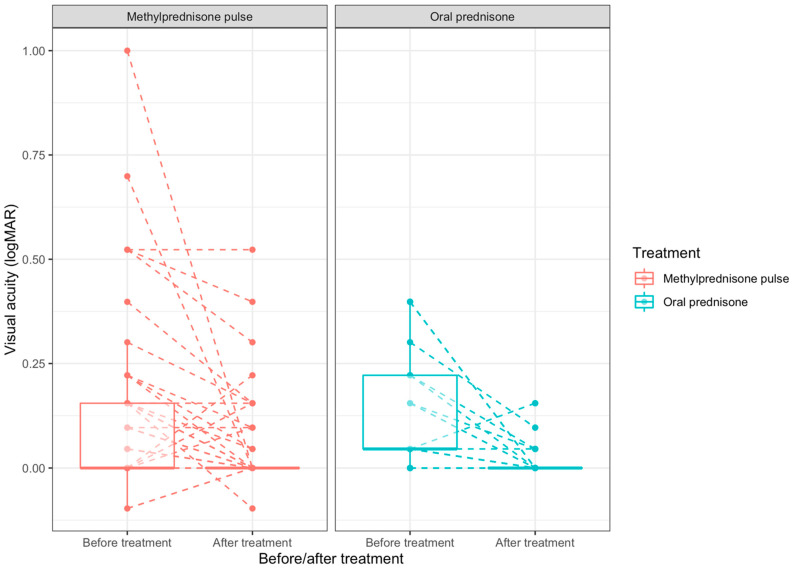
BCVA distribution among the studied groups and progression before/after treatment. BCVA: Best-corrected visual acuity. Median [IQR] BCVA before treatment in the methylprednisolone group: 0 [0–0.155]; Median [IQR] BCVA before treatment in the prednisone group: 0.0458 [0.0458–0.222]; Median [IQR] BCVA before treatment in the whole population: 0.0458 [0–0.155]; Median [IQR] BCVA after treatment in the methylprednisolone group: 0 [0–0]; Median [IQR] BCVA after treatment in the prednisone group: 0 [0–0]; Median [IQR] BCVA after treatment in the whole population: 0 [0–0].

The overall mean ± SD final visual acuity was 0.0365 ± 0.0937; it was 0.0435 ± 0.107 in the methylprednisolone group and 0.0185 ± 0.0403 in the prednisone group. There was a significant BCVA improvement from baseline to final visit for the whole population (mean difference: −0.086 LogMAR 95% CI [−0.126; −0.046]; *p* < 0.001), for the methylprednisolone group (−0.069 LogMAR 95% CI [−0.116; −0.022]; *p* = 0.005), and for the oral prednisolone group (−0.128 LogMAR 95% CI [−0.203; −0.052]; *p* = 0.001). There was no significant difference in BCVA improvement between the methylprednisolone and prednisone groups (mean difference of BCVA progression between groups: 0.059, 95% CI [−0.030; 0.147]; *p* = 0.201; Table 2). The median BCVA progression and BCVA distribution before and after treatment is represented in Figure 2. As can be seen, the distribution of initial BCVA differed between the two groups, even though the mean initial BCVA did not differ significantly (Table 1). Patients with severely impaired visual function at baseline tended to highly recover (Figure 2).

### 2.3. Inflammation Control

**Figure 3:**
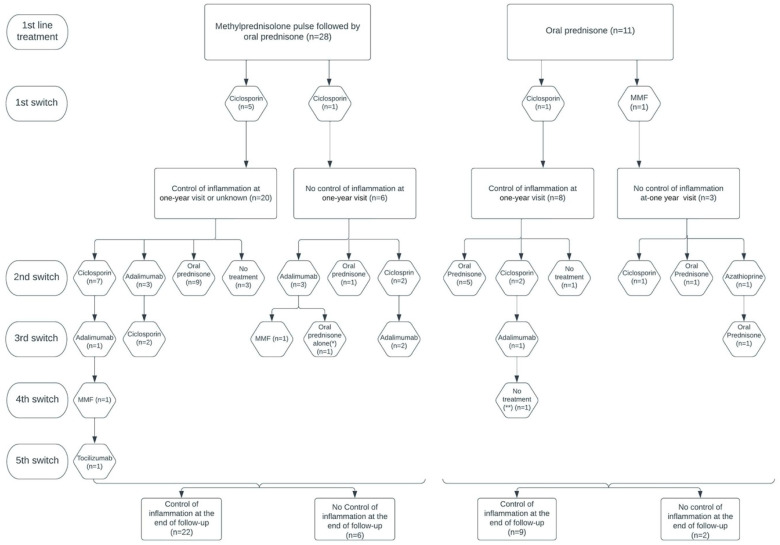
Treatment regimen and changes during follow-up. *, ** Two patients of the methylprednisolone group did not undergo the one-year visit.

**Figure 4:**
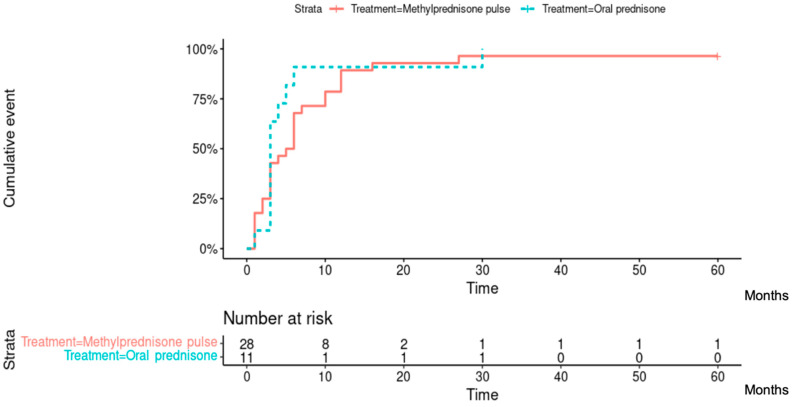
Cumulative incidence curves of inflammation control according to treatment group. Median [IQR] time to inflammation control (months) in the methylprednisolone group: 5 [2–10]; Median [IQR] time to inflammation control (months) in the prednisone group: 3 [3–5].

Among the 37 patients evaluated at 1 year after treatment onset (two patients did not receive the 1-year assessment), 28 (75.7%) had reached inflammation control, corresponding to 20/26 (76.9%) patients from the methylprednisolone group and 8/11 (72.7%) patients from the prednisone group. At the end of follow-up, 31/39 (79.5%) patients had reached inflammation control, corresponding to 22/28 (78.6%) patients from the methylprednisolone group and 9/11 (81.8%) patients from the prednisone group (Figure 3). A total of 38/39 (97.4%) patients had reached inflammation control at some point during their follow-up, corresponding to 27/28 (96.4%) patients from the methylprednisolone group and 11/11 (100%) patients from the prednisone group. There was no difference between the two groups of patients in the time required to control inflammation (Hazard ratio [95% CI]: 0.719 [0.350; 1.477]; *p* = 0.40). The median [IQR] time to inflammation control was 5 [2,3,4,5,6,7,8,9,10] months in the methylprednisolone group and 3 [3,4,5] months in the prednisone group (Figure 4).

In the methylprednisolone group, 13/28 (46.4%) patients were treated exclusively with intravenous cortisone with oral relay and 12/13 (92.3%) had reached inflammation control at their last assessment. At last check, 3/13 (23.1%) patients were weaned off cortisone; their mean (range) follow-up duration without treatment was 27.3 (18–34) months. Among these 13 patients, the mean (range) cortisone dose at the end of follow-up was 3.5 (0–9) mg/day.

In the prednisone group, 6/11 (54.5%) patients were treated exclusively with prednisone and among them, 6/6 (100%) had reached inflammation control at their last assessment. At last check, one (16.6%) patient was weaned off cortisone (18 months of follow-up without treatment). Among these six patients, the mean (range) cortisone dose at the end of follow-up was 3.6 (0–10) mg/day.

A total of three patients in the cohort were considered in remission at the end of follow-up (two in the methylprednisolone group, one in the prednisone group), requiring no treatment for at least 18 months. Those patients were followed for a mean (range) duration of 25 (18–34) months without treatment and did not display any sign of relapse during this period. One patient from the methylprednisolone group relapsed after 18 months of monitoring without treatment.

### 2.4. Relapse Rate and Time to Relapse

The number of relapses and time to relapse was analyzed for 38 patients, as one patient in the methylprednisolone group never achieved inflammation control (60 months follow-up). A total of 28/38 (73.7%) patients relapsed at least once, corresponding to 22/27 (81.5%) patients from the methylprednisolone group and 6/11 (54.5%) patients from the prednisone group. A total of 15/38 (39.5%) patients relapsed at least twice, corresponding to 11/27 (40.7%) patients from the methylprednisolone group and 4/11 (36.4%) patients from the prednisone group).

The overall annualized relapse rate was 0.330 person/year. The methylprednisolone and the prednisone groups had an annualized relapse rate of 0.344 person/year and 0.292 person/year, respectively. There was no statistically significant difference between these two groups (RR = 1.117, 95% CI [0.508; 2.729]; *p* = 0.704).

The time to relapse was assessed in the 28 relapsing patients (who experienced at least one relapse). The median [IQR] time to relapse was 15 [6.25–46.5] months in the methylprednisolone group and 11 [8.5–37.5] months in the prednisone group (*p* = 0.93).

### 2.5. Use of Immunosuppressive Therapy

In the methylprednisolone group, 15/28 (53.6%) patients received immunosuppressive therapy (IST) during their follow-up and 10/15 (66.7%) had reached inflammation control at the end of follow-up. Among these 15 patients, the mean (range) dose of cortisone at the end of follow-up was 10.1 (2–40) mg/day. At the end of follow up, 14/15 (93.3%) patients were still under IST: 8/14 (57.1%) were treated with ciclosporin, 4/14 (28.6%) with adalimumab, 1/14 (7.1%) with mofetilmycophenolate (MMF), and 1/14 (7.1%) with tocilizumab (Figure 3). The reasons for initiating IST were recurrent relapses and/or high-dose corticosteroid dependence (>7.5 mg/day) for 11/15 (73.3%) patients, refractory IME for 3/15 (20.0%) patients, and inclusion in a clinical trial for 1/15 (6.7%) patient.

In the prednisone group, 5/11 (45.5%) patients received IST during follow-up and 3/5 (60.0%) had reached inflammation control at the end of follow-up. Among these five patients, the mean (range) cortisone dose at the end of follow-up was 5.6 (5–8) mg/day. At the end of follow-up, two (40.0%) of them were still under IST therapy and both were treated with ciclosporin. Two (40.0%) patients were treated with prednisone alone (one because of IST refusal and the other because of clinical improvement allowing the IST to be discontinued). One (20.0%) patient was administered no treatment (Figure 3). The reasons for initiating immunosuppressive treatment were iterative relapses and/or high-dose corticosteroids dependence (>7.5 mg/day) for 2/5 (40.0%) patients and refractory IME for 3/5 (60.0%) patients.

Overall, among the 20 patients who were administered IST, the reasons for initiating treatment were recurrent relapses and/or high dose corticosteroids dependence for 13 (65.0%) patients, refractory IME for six (30.0%) patients, and inclusion in a trial protocol for one (5.0%) patient. There was no statistically significant difference in the use of IST between the methylprednisolone and prednisone groups (OR = 0.73, 95% CI [0.14; 3.65]; *p* = 0.73).

### 2.6. Inflammatory Macular Edema

Inflammatory macular edema (IME) was present in 13 (33.3%) patients at inclusion and involved only one eye for six of them (46.2%). Treatment with intravitreal injections in addition to background cortisone therapy was a first-line choice treatment for unilateral refractory IME (intravitreal injections were also used for bilateral edema when the IME was highly asymmetric, or when patients refused to switch to a second line immunosuppressive therapy). However, 8/13 (61.5%) patients received one or more intravitreal injections of corticosteroids (dexamethasone implant or intra vitreal injection of triamcinolone), usually allowing the control of the IME in association with systemic cortisone anti-inflammatory treatment.

During their follow-up, 4/13 (30.8%) patients had at least four recurrences of IME, with no other sign of clinically or angiographically active inflammation, requiring the use of IST (none of these patients had a surgical background suggesting an Irvine–Gass syndrome could be implicated).

A total of 8/13 (61.5%) patients presenting IME at baseline or recurrent required the initiation of an IST during their follow-up, in order to reduce the IME. A total of 9/13 (69.2%) patients relapsed at least once during their follow-up, 6/13 (46.2%) relapsed several times, and 1/13 (7.7%) patient never reached inflammation control (due to refractory IME) before loss to follow-up (60 months follow-up).

At the end of follow-up, 4/13 (30.8%) patients had not reached inflammatory control, one (7.7%) of them due to persistent IME. As a result, 9/13 (69.2%) patients in this subgroup of interest had reach inflammatory control at the end of follow up in comparison to 22/26 (84.6%) patients who did not present any IME during their follow-up (OR = 0.42, 95% CI [0.06; 2.78]; *p* = 0.40).

### 2.7. Side Effects

Side effects due to corticotherapy concerned 37/39 (94.9%) patients treated, but none required treatment discontinuation. The most frequent side effects were Cushingoid features (n = 12), nervosity and sleeping disorders (n = 12), osteopenia/non-fractural osteoporosis (n = 9), and hypertension (n = 6). Among patients treated with IST we did not observe any side effect for those treated with azathioprine and MMF. Among the 17 patients treated with ciclosporin, five (29.4%) presented nephrotoxicity, which was irreversible for one (5.9%) of them, four (23.5%) patients developed high blood pressure, and five (29.4%) patients had hirsutism. A total of four (23.5%) patients developed serious side effects requiring the discontinuation of this treatment. Among them, one patient had a severe fungal infection. Finally, adalimumab was often used as a second- or third-line therapy and three patients experienced serious side effects requiring its discontinuation (induced Lupus, major hepatic cytolysis, and development of a prostatic tumor; Table 3).

## 3. Discussion

Studies investigating the effects of systemic corticotherapy alone during the course of BSCR are rare [21]. We investigated the prolonged corticosteroid therapy treatment strategy, which resulted in inflammation control in almost half of patients with BSCR. This control was maintained with low doses of cortisone, usually less than 5 mg daily. When control with corticosteroids alone required high doses (>7.5 mg/day) or could not be achieved, the use of an IST achieved control in almost all cases. We did not find any difference between the group of patients initially treated with methylprednisolone and the group of patients initially treated with prednisone alone regarding visual acuity improvement, time to reach inflammation control, number of relapses, time to relapse, and use of IST. Of note, the patients included in our study presented demographic characteristics consistent with those of other studies on BSCR, especially for the prevalence of vasculitis, IME, and papillitis [17,26].

Contrary to what has been found by several recent study [27,28], we found a statistically significant improvement in BCVA before/after treatment, overall and in the two groups of interest. It should be noted, however, that although the mean initial BCVA did not differ between these two groups, there was a different distribution of patients between subgroups: our results suggest a greater visual improvement for patients with severely impaired initial BCVA (<20/40 Snellen, Log MAR 0.30) in the methylprednisolone group, supporting our practice of administering corticosteroids loading doses to these patients. It has been previously demonstrated that an early and intense initial treatment, as well as a prolonged treatment, allows a better preservation of the visual function [15,16,29]. As stated in many studies [5,13,14,26], visual acuity only deteriorates late during the course of BSCR while visual blur and discomfort are frequent complaints of patients that considerably impair their quality of life [30]. Alterations of the BCVA are most often the consequence of an IME [14,26], which has itself recently been singled out as a possible factor of treatment resistance [31]. As almost 40% of patients from the methylprednisolone group had IME at inclusion, this may have contributed to limit visual recovery in this group, especially since about half of the patients with initial IME were uncontrolled or experienced relapsed by the end of follow-up. Although the BCVA is not the most reliable indicator of clinical improvement under treatment [17], it remains an easily accessible and exploitable indicator of the patients functional improvement [30] and of the improvement in macular and/or vitreous inflammatory state. In addition, it is one of the most frequently used outcomes in therapeutic comparison studies in BSCR [16,32], justifying the relevance of its use in our study.

Contrary to what several authors found almost 20 years ago [14], progress in therapeutics and early diagnosis have led to a considerable improvement in the visual prognosis. BSCR no longer appears as an inevitably blinding condition, as evidenced by the progression of the median Snellen visual acuity in our study, close to 20/20 at the end of follow-up (logMar 0). It is reasonable to assume that the present cohort included a few “benign” BSCR cases [5], detected and treated early with systemic corticosteroids, which may partly explain these good BCVA results.

Birdshot’s patients are inherently different, depending on the timepoint in their disease at presentation, whether the inflammation is more choroidal, retinal, or symmetrical, and whether there is an IME or not. As a matter of fact, our treatment habits differed according to initial BCVA and inflammation intensity at baseline. Another important element in the initial management of our patient was the therapeutic habits of each practitioner, our patients being recruited from two different centers, with different therapeutic habits regarding initial loading doses. Patients with a severely impaired initial BCVA and a major inflammation at presentation were more likely to be treated initially with methylprednisolone. It is, therefore, hard to conclude that methylprednisolone may not be helpful in the management of BSCR.

Almost all patients had reached inflammation control during follow-up, which is consistent with other studies investigating BSCR [27], and the median time to reach this control was less than five months in both groups. Although control is often reached quickly, relapses are common in BSCR when corticosteroid therapy is tapered [33,34]. The pattern of relapse in BSCR has been studied recently by Crowell et al. [33] in a cohort study including twice as many patients as ours, and reporting an annualized relapse rate of 0.240 person/year, which is similar to the relapse rate found herein. Crowell et al. have reported a 25% remission rate by four years of follow-up, as drug-free remission was defined by an inactive disease off all medications for ≥3 months. The median follow-up duration in our study was almost twice as long, and we considered an 18-month drug-free period without any sign of inflammation to presume patients to be cured. Less than 10% of our patients were considered to be in remission at the end of follow-up and the mean drug-free duration was 25 months. Furthermore, nearly a fifth of patients were experiencing a relapse or had not reach control at the end of follow up. In our experience and as demonstrated in many studies [31,33], BSCR is a condition requiring prolonged treatment with a very slow tapering in corticosteroid therapy, sometimes with the long-term maintenance of a low dose of cortisone if well tolerated (>30 months according to Maleki et al. [28]). As relapses occurred in nearly a third of patients per year, a delay of three months does not allow us to consider a patient in remission, thereby explaining the major difference in results between the study issued by Crowell et al. and ours.

Although some studies have found IME as a poor prognostic factor in the context of BSCR [31], our data did not show a significant difference in inflammation control at the end of follow-up between patients with and without IME. Similarly, we did not find more relapses in this subgroup of interest compared to the entire cohort.

Our results support that the long-term preservation of the visual function and ocular inflammation control may be achievable with low-dose systemic corticosteroids alone for half of the patients with BSCR. In contrast to our findings, some authors have considered that “unacceptably high” maintenance doses are required to control inflammation in BSCR patients [16] advocating for the rapid use of IST in these patients [15,17].

Although steroid side effects are frequent, they are generally not serious and diminish with the treatment tapering to doses <7.5 mg/day [33]. Some clinical characteristics of the disease, such as IME, seem to require more a frequent use of IST, which is consistent with the data of Maleki et al. [31]. In addition, some authors have already demonstrated the effectiveness of IST in preventing IME, in comparison with corticosteroid therapy [17]. The effectiveness of DMARDs and new biotherapies has been widely demonstrated in the field of non-infectious posterior uveitis [33], but it should be remembered that these treatments are also prone to serious adverse effects, as shown in our study. As almost half of BSCR patients can reach control with a low-dose of prednisone alone, we consider that these treatments, as for other chronic non-infectious uveitis, should be considered for patients who respond inadequately to corticosteroids, for whom corticosteroid treatment is inappropriate, or when a corticosteroid sparing is necessary [33].

Our study has several limitations. As BSCR is a rare disease, the sample size was small despite a recruitment over more than 15 years in two tertiary centers specialized in ocular inflammation. Our study was conducted retrospectively, and many patients were excluded from the analysis due to the lack of usable data/several missing data, which may constitute a selection bias specific to retrospective studies. Unfortunately and contrary to other studies [16], we were not able to assess the prevalence of ophthalmological complications related to BSCR or its treatment (ERM, cataract, glaucoma…) due to the lack of data, or because it was difficult to distinguish the component involved in the occurrence of these complications.

## 4. Conclusions

We confirmed the trend already observed in a study previously published by our research team, investigating inflammatory control and the effectiveness of low-dose corticosteroid therapy in BSCR [21]. Initial loading doses did not appear to significantly reduce the time needed to reach inflammation control or the number of relapses, nor did they improve the final visual prognosis. However, as the two groups are not comparable, if the initial BCVA is poor (mainly due to inflammatory macular edema), we suggest a loading dose that could improve the visual prognosis rapidly and durably. When corticosteroid therapy is insufficient, the introduction of an IST allows inflammation control in most patients. BSCR is a chronic condition for which only about 10% of patients can be considered as cured in the long term, in our experience. Although many studies have been conducted in recent years, there is currently no consensus on the optimal treatment modalities and duration of treatment for BSCR. Further investigations such as prospective randomized studies are needed to address these points.

## Figures and Tables

**Table 1:** Patients and disease characteristics at inclusion.

		Total (n = 39)	Methylprednisolone Pulse (n = 28)	Oral Prednisone (n = 11)
**Demographics**				
	**Gender**			
	Female [mean (%)]	20 (51.3)	14 (50)	6 (54.5)
	**Ethnicity**			
	Caucasian [mean (%)]	39 (100)	28 (100)	11 (100)
	**Laterality**			
	Bilateral [mean (%)]	38 (97.4)	28 (100)	10 (90.9)
	**Mean Age [EV]**	50 [26–79]	51 [27–79]	47.4 [26–59]
	**Mean Duration of follow-up [EV]**	78.8 [23–186]	75.9 [23–171]	86.3 [30–186]
**initial findings**				
	**HLA A-29 positivity [mean (%)]**	39 (100)	28 (100)	11 (100)
	Initial BCVA LogMar (*) [mean (SD)]	0.122 (0.192)	0.113 (0.206)	0.146 (0.151)
	**Macular oedema [mean (%)]**	13 (33.3)	11 (39.3)	2 (18.2)
	Bilatéral [mean (%)]	7 (17.9)	5 (17.9)	2 (18.2)
	**Inflammation’s location [mean (%)]**			
	Intermediate + posterior [mean (%)]	31 (79.4)	22 (78.6)	9 (81.9)
	Posterior only [mean (%)]	6 (15.4)	5 (17.9)	1 (9.1)
	pan-uveitis [mean (%)]	2 (5.1)	1 (3.6)	1 (9.1)
	**Clinical retinal vasculitis (1) [mean (%)]**	22 (56.4)	20 (71.4)	2 (18.2)
	Bilateral [mean (%)]	18 (46.2)	16 (57.1)	2 (18.2)
	**Hypofluorescent ICG-spots [mean (%)]**	39 (100)	28 (100)	11 (100)
	**Papillar oedema [mean (%)]**	22 (56.4)	15 (53.6)	7 (63.6)
	Bilateral [mean (%)]	20 (51.3)	15 (53.6)	5 (45.4)
**Motivation for treatment**				
	**Visual loss [mean (%)]**	14 (35.9)	12 (42.9)	2 (18.2)
	**Visual field distorsion [mean (%)]**	3 (7.3)	2 (7.1)	1 (9.1)
	**Myodesopsia [mean (%)]**	10 (24.4)	8 (28.6)	2 (18.2)
	**Other/unknown [mean (%)]**	15 (38.5)	11 (39.3)	4 (36.4)
**Main comorbidities**				
	**Ophtalmological background [mean (%)]**			
	Cataract surgery [mean (%)]	4 (10.3)	3 (10.7)	1 (9.1)
	Retinal detachment [mean (%)]	3 (7.3)	2 (7.1)	1 (9.1)
	Endophtalmitis [mean (%)]	1 (2.4)	1 (3.6)	0 (0)
	Amblyopia [mean (%)]	2 (4.9)	2 (7.1)	0 (0)
	Idiopathic Intracranial Hypertension [mean (%)]	1 (2.4)	1 (3.6)	0 (0)
	**Systemic background**			
	Systemic hypetension [mean (%)]	12 (30.1)	8 (28.6)	4 (36.4)
	Dyslipidemia [mean (%)]	7 (17.9)	5 (17.9)	2 (18.2)
	Diabete mellitus [mean (%)]	2 (4.9)	0 (0)	2 (18.2)
	Hypothyroidism [mean (%)]	4 (9.8)	1 (3.6)	3 (27.3)
	Tumor (2) [mean (%)]	5 (12.8)	3 (10.7)	2 (18.2)
**Follow-up**				
	**Mean Duration of follow-up [EV]**	78.8 [23–186]	75.9 [23–171]	86.3 [30–186]

BSCR: Birdshot chorioretinopathy; BCVA: best-corrected visual acuity; EV: extremes values; ICGA: Indocyanine green angiography; SD: standard deviation. (1) Fluorescein angiography was not available for all patients at inclusion unlike fundus examination, explaining why we preferred to present the clinical assessment for vasculitis. (2) Three patients from the methylprednisolone group had a tumor background at inclusion: one had a history of pituitary adenoma, one of breast cancer, and one of focal nodular hyperplasia of the liver (FNH). Two patients from the prednisone group had a background of breast cancer as well for the first one and a pancreatic IPMN (Intraductal Papillary Mucinous Neoplasm) for the second one. (*) BCVA at inclusion did not differ significantly between the methylprednisolone group and the initial prednisone group (−0.034, 95% CI [−0.110; 0.043]; *p* = 0.389).

**Table 2:** Best corrected visual acuity (BCVA; in LogMAR) evaluation and comparison between groups.

Group	Before Treatment	After Treatment	Before/After Comparison	*p*	Interaction	*p*
Total (n = 75) [Mean (SD)]	0.122 (0.192)	0.0365 (0.0937)	−0.086 (−0.126; −0.046)	<0.001		
M-Pred pulse (n = 54) [Mean (SD)]	0.113 (0.206)	0.0435 (0.107)	−0.069 (−0.116; −0.022)	0.005	0.059 (−0.030; 0.147)	0.201
Oral Pred (n = 21) [Mean (SD)]	0.146 (0.151)	0.0185 (0.0403)	−0.128 (−0.203; −0.052)	0.001		

CI: confidence interval; M-Pred: methylprednisolone; Pred: prednisolone; SD: standard deviation.

**Table 3:** Side effects according to the different treatments.

Side Effects	Corticosteroid (n = 39)	Ciclosporin (n = 17)	Azathioprine (n = 1)	Mycophénolate Mofetil (n = 3)	Adalimumab (n = 10)
**Neuromuscular**					
Nervosity and sleeping disorders [mean (%)]	12 (30.8)	0 (0)	0 (0)	0 (0)	0 (0)
Disabling tremor [mean (%)]	2 (5.1)	0 (0)	0 (0)	0 (0)	0 (0)
Cramps [mean (%)]	3 (7.7)	1 (5.3)	0 (0)	0 (0)	0 (0)
Numbness [mean (%)]	0 (0)	1 (5.3)	0 (0)	0 (0)	0 (0)
**Osteoarticular**					
Osteopenia and Osteoporosis [mean (%)]	9 (23.1)	0 (0)	0 (0)	0 (0)	0 (0)
**Endocrinological**					
Oedema and Hypercorticism [mean (%)]	12 (30.8)	0 (0)	0 (0)	0 (0)	0 (0)
Diabete mellitus [mean (%)]	2 (5.1)	0 (0)	0 (0)	0 (0)	0 (0)
Hirsutism [mean (%)]	4 (10.2)	5 (26.3)	0 (0)	0 (0)	0 (0)
Acne and folliculitis [mean (%)]	6 (15.4)	0 (0)	0 (0)	0 (0)	0 (0)
**Dermatological**					
Skin fragility [mean (%)]	3 (7.7)	0 (0)	0 (0)	0 (0)	0 (0)
Flushes [mean (%)]	0 (0)	1 (5.3)	0 (0)	0 (0)	0 (0)
Gingival Hypertrophy [mean (%)]	0 (0)	3 (15.8)	0 (0)	0 (0)	0 (0)
**Digestive**					
Peptic Ulcer [mean (%)]	3 (7.7)	0 (0)	0 (0)	0 (0)	0 (0)
Steatosis [mean (%)]	1 (2.6)	0 (0)	0 (0)	0 (0)	0 (0)
Hepatic cytolysis [mean (%)]	0 (0)	0 (0)	0 (0)	0 (0)	1 (10)
**Cardio-vascular**					
Hypertension [mean (%)]	5 (12.8)	4 (21.1)	0 (0)	0 (0)	0 (0)
Dyslipidemia [mean (%)]	1 (2.6)	0 (0)	0 (0)	0 (0)	0 (0)
**Renal**					
Kidney failure [mean (%)]	0 (0)	5 (26.3)	0 (0)	0 (0)	0 (0)
**Infectious**					
Zoster [mean (%)]	2 (5.1)	1 (5.3)	0 (0)	0 (0)	0 (0)
Infection [mean (%)]	2 (5.1) *	1 (5.3) **	0 (0)	0 (0)	0 (0)
**Ophtalmologic**					
Induced Ocular Hypertonia [mean (%)]	3 (7.7)	0 (0)	0 (0)	0 (0)	0 (0)
**Others**					
Hyper-leukocytosis [mean (%)]	3 (7.7)	0 (0)	0 (0)	0 (0)	0 (0)
Anemia [mean (%)]	0 (0)	1 (5.3)	0 (0)	0 (0)	0 (0)
Ashtenia [mean (%)]	0 (0)	0 (0)	0 (0)	0 (0)	0 (0)
Induced Lupus [mean (%)]	0 (0)	0 (0)	0 (0)	0 (0)	1 (10)
Carcinoma [mean (%)]	0 (0)	0 (0)	0 (0)	0 (0)	1 (10) ***

(*) Both cases of infection in patients treated with corticotherapy were non-serious pulmonary infection; (**) Serious fungal infection requiring hospitalization and discontinuation of this treatment; (***) Prostatic adenocarcinoma.

## References

[1] Jones N.P. (2015). The Manchester Uveitis Clinic: The first 3000 patients, 2: Uveitis Manifestations, Complications, Medical and Surgical Management. Ocul. Immunol. Inflamm..

[2] Gelfman S., Monnet D., Ligocki A.J., Tabary T., Moscati A., Bai X., Freudenberg J., Cooper B., Kosmicki J.A., Wolf S. (2021). ERAP1, ERAP2, and Two Copies of HLA-Aw19 Alleles Increase the Risk for Birdshot Chorioretinopathy in HLA-A29 Carriers. Investig. Ophthalmol. Vis. Sci..

[3] Herbort C.P., Neri P., Papasavvas I. (2021). Clinicopathology of non-infectious choroiditis: Evolution of its appraisal during the last 2-3 decades from «white dot syndromes» to precise classification. J. Ophthalmic. Inflamm. Infect..

[4] Touhami S., Fardeau C., Vanier A., Zambrowski O., Steinborn R., Simon C., Tezenas du Moncel S., Bodaghi B., Lehoang P. (2016). Birdshot Retinochoroidopathy: Prognostic Factors of Long-term Visual Outcome. Am. J. Ophthalmol..

[5] Lages V., Skvortsova N., Jeannin B., Gasc A., Herbort C.P. (2019). Low-grade «benign» birdshot retinochoroiditis: Prevalence and characteristics. Int. Ophthalmol..

[6] Minos E., Barry R.J., Southworth S., Folkard A., Murray P.I., Duker J.S., Keane P.A., Denniston A.K. (2016). Birdshot chorioretinopathy: Current knowledge and new concepts in pathophysiology, diagnosis, monitoring and treatment. Orphanet J. Rare Dis..

[7] Ryan S.J., Maumenee A.E. (1980). Birdshot retinochoroidopathy. Am. J. Ophthalmol..

[8] Gass J.D. (1981). Vitiliginous chorioretinitis. Arch. Ophthalmol. Chic. Ill..

[9] Papadia M., Pavésio C., Fardeau C., Neri P., Kestelyn P.G., Papasavvas I., Herbort C.P. (2021). HLA-A29 Birdshot Retinochoroiditis in Its 5th Decade: Selected Glimpses into the Intellectual Meanderings and Progresses in the Knowledge of a Long-Time Misunderstood Disease. Diagnostics.

[10] Herbort C.P., Pavésio C., LeHoang P., Bodaghi B., Fardeau C., Kestelyn P., Neri P., Papadia M. (2017). Why birdshot retinochoroiditis should rather be called “HLA-A29 uveitis”?. Br. J. Ophthalmol..

[11] Cao J.H., Silpa-Archa S., Freitas-Neto C.A., Foster C.S. (2016). Birdshot chorioretinitis lesions on indocyanine green angiography as an indicator of disease activity. Retina.

[12] Standardization of Uveitis Nomenclature (SUN) Working Group (2021). Classification Criteria for Birdshot Chorioretinitis. Am. J. Ophthalmol..

[13] Priem H.A., Oosterhuis J.A. (1988). Birdshot chorioretinopathy: Clinical characteristics and evolution. Br. J. Ophthalmol..

[14] Rothova A., Berendschot T.T.J.M., Probst K., van Kooij B., Baarsma G.S. (2004). Birdshot chorioretinopathy: Long-term manifestations and visual prognosis. Ophthalmology.

[15] Becker M.D., Wertheim M.S., Smith J.R., Rosenbaum J.T. (2005). Long-term follow-up of patients with birdshot retinochoroidopathy treated with systemic immunosuppression. Ocul. Immunol. Inflamm..

[16] Kiss S., Ahmed M., Letko E., Foster C.S. (2005). Long-term follow-up of patients with birdshot retinochoroidopathy treated with corticosteroid-sparing systemic immunomodulatory therapy. Ophthalmology.

[17] Thorne J.E., Jabs D.A., Peters G.B., Hair D., Dunn J.P., Kempen J.H. (2005). Birdshot retinochoroidopathy: Ocular complications and visual impairment. Am. J. Ophthalmol..

[18] Calvo-Río V., Blanco R., Santos-Gómez M., Díaz-Valle D., Pato E., Loricera J., Gonzalez-Vela M.C., Demetrio-Pablo R., Hernandez J.L., Gonzalez-Gay M.A. (2017). Efficacy of Anti-IL6-Receptor Tocilizumab in Refractory Cystoid Macular Edema of Birdshot Retinochoroidopathy Report of Two Cases and Literature Review. Ocul. Immunol. Inflamm..

[19] Huis Het Veld P.I., van Asten F., Kuijpers R.W.A.M., Rothova A., de Jong E.K., Hoyng C.B. (2019). Adalimumab therapy for refractory birdshot chorioretinopathy. Retina.

[20] Géhl Z., Szepessy Z., Nagy Z.Z. (2021). Ophthalmic use of TNFα inhibitor: Adalimumab treatment in uveitis. Orv. Hetil..

[21] Hafidi M., Loria O., Kodjikian L., Denis P., Ferrand M.R., Broussolle C., Seve P. (2017). Efficacy of Methylprednisolone Pulse Followed by Oral Prednisone in Birdshot Chorioretinopathy. Ocul. Immunol. Inflamm..

[22] Nussenblatt R.B., Palestine A.G., Chan C.C., Roberge F. (1985). Standardizatlon of Vitreal inflammatory Activity in Intermediate and Posterior Uveitis. Ophthalmology.

[23] Hellmich B., Agueda A., Monti S., Buttgereit F., de Boysson H., Brouwer E., Cassie R., Cid M.C., Dasgupta B., Dejaco C. (2020). 2018 Update of the EULAR recommendations for the management of large vessel vasculitis. Ann. Rheum. Dis..

[24] Bienvenu B., Ly K.H., Lambert M., Agard C., André M., Benhamou Y., Bonnotte B., de Boysson H., Espitia O., Fau G. (2016). Management of giant cell arteritis: Recommendations of the French Study Group for Large Vessel Vasculitis (GEFA). Rev. Med. Interne.

[25] Comander J., Loewenstein J., Sobrin L. (2011). Diagnostic testing and disease monitoring in birdshot chorioretinopathy. Semin. Ophthalmol..

[26] Monnet D., Brézin A.P., Holland G.N., Yu F., Mahr A., Gordon L.K., Levinson L.D. (2006). Longitudinal cohort study of patients with birdshot chorioretinopathy. I. Baseline clinical characteristics. Am. J. Ophthalmol..

[27] You C., Lasave A.F., Kubaisi B., Syeda S., Ma L., Wai K.C.K., Hernandez Diaz M., Walsh M., Stephenson A., Montieth A. (2020). Long-term outcomes of systemic corticosteroid-sparing immunomodulatory therapy for Birdshot Retinochoroidopathy. Ocul. Immunol. Inflamm..

[28] da Fonsêca M.L.G., Vianna R.N.G., Rocha A.C.H., Casella A.M.B., Cialdini A., Muccioli C., da Costa D.S., Lucena D.R., Vasconcelos-Santos D.V., Morizot E. (2022). Birdshot retinochoroiditis in Brazil: A multicenter review of 40 patients. Int. J. Retin. Vitr..

[29] Knecht P.B., Papadia M., Herbort C.P. (2014). Early and sustained treatment modifies the phenotype of birdshot retinochoroiditis. Int. Ophthalmol..

[30] Pohlmann D., Barth A., Macedo S., Pleyer U., Winterhalter S., Albayrak Ö. (2020). The impact of impending/onset of vision loss on depression, anxiety, and vision-related quality of life in Birdshot-Retinochoroiditis and Serpiginous Choroiditis. PLoS ONE.

[31] Maleki A., Look-Why S., Manhapra A., Asgari S., Garcia C.M., Al-Dabbagh A., Tsang C., Chang P.Y., Anesi S.D., Foster C.S. (2022). Birdshot Chorioretinopathy: Resistant versus Responsive. Ocul. Immunol. Inflamm..

[32] Leclercq M., Langlois V., Girszyn N., Le Besnerais M., Benhamou Y., Levesque H., Muraine M., Gueudry J. (2020). Comparison of conventional immunosuppressive drugs versus anti-TNF-α agents in non-infectious non-anterior uveitis. J. Autoimmun..

[33] Crowell E.L., France R., Majmudar P., Jabs D.A., Thorne J.E. (2022). Treatment Outcomes in Birdshot Chorioretinitis: Corticosteroid Sparing, Corticosteroid Discontinuation, Remission, and Relapse. Ophthalmol. Retin..

[34] Maleki A., Look-Why S., Manhapra A., Asgari S., Philip A.M., Chang P.Y., Anesi S.D., Foster C.S. (2023). Late recurrence in birdshot chorioretinopathy. Can. J. Ophthalmol..

